# Equine cervical intervertebral disc degeneration is associated with location and MRI features

**DOI:** 10.1111/vru.12794

**Published:** 2019-07-28

**Authors:** Stefanie Veraa, Wilhelmina Bergmann, Inge D. Wijnberg, Willem Back, Hans Vernooij, Mirjam Nielen, Antoon‐Jan M. van den Belt

**Affiliations:** ^1^ Division of Diagnostic Imaging, Faculty of Veterinary Medicine Utrecht University Utrecht The Netherlands; ^2^ Department of Pathobiology, Faculty of Veterinary Medicine Utrecht University Utrecht The Netherlands; ^3^ Department of Equine Sciences, Faculty of Veterinary Medicine Utrecht University Utrecht The Netherlands; ^4^ Department of Surgery and Anaesthesiology of Domestic Animals, Faculty of Veterinary Medicine Ghent University Merelbeke Belgium; ^5^ Department of Farm Animal Health, Faculty of Veterinary Medicine Utrecht University Utrecht The Netherlands

**Keywords:** annulus fibrosus, disc disease, nucleus pulposus, spinal column

## Abstract

Morphology of the equine cervical intervertebral disc is different from that in humans and small companion animals and published imaging data are scarcely available. The objectives of this exploratory, methods comparison study were (a) to describe MRI features of macroscopically nondegenerated and degenerated intervertebral discs (b) to test associations between spinal location and macroscopic degeneration or MRI‐detected annular protrusion and between MRI‐detected annular protrusion and macroscopic degeneration, and (c) to define MRI sequences for characterizing equine cervical intervertebral disc degeneration. Ex vivo MRI of intervertebral discs was performed in 11 horses with clinical signs related to the cervical region prior to macroscopic assessment. Mixed‐effect logistic regression modeling included spinal location, MRI‐detected annular protrusion, and presence of macroscopic degeneration with “horse” as random effect. Odds ratio and 95% confidence interval were determined. Reduced signal intensity in proton density turbo SE represented intervertebral disc degeneration. Signal voids due to presence of gas and/or hemorrhage were seen in gradient echo sequences. Presence of macroscopic intervertebral disc degeneration was significantly associated with spinal location with odds being higher in the caudal (C5 to T1) versus cranial (C2 to C5) part of the cervical vertebral column. Intervertebral discs with MRI‐detected annular protrusion grades 2‐4 did have higher odds than with grade 1 to have macroscopic degeneration. It was concluded that MRI findings corresponded well with gross macroscopic data. Magnetic resonance imaging of the equine cervical intervertebral disc seems to be a promising technique, but its potential clinical value for live horses needs to be explored further in a larger and more diverse population of horses.

## 1 INTRODUCTION

Intervertebral disc disease is a well‐known clinical entity in man and small companion animals and studies on normal anatomy, composition, and pathological conditions of the intervertebral disc are manifold.^1^, ^2^, ^3^ In horses, the situation is different. The equine intervertebral disc has previously been described to have a different composition compared to that of most other mammals and to consist almost entirely of fibrous to fibrocartilaginous elements without a well‐delineated nucleus pulposus.^4^, ^5^ However, new data do support the presence of a cartilaginous nucleus pulposus and fibrous annulus fibrosus similar to that in other mammals.^6^ Intervertebral disc degeneration in the equine cervical spine has been characterized macroscopically by fibrillation, in severe cases accompanied by yellow discoloration and cleft formation and a gross degeneration grading scheme has been proposed.^4^, ^6^ Several case reports describe equine intervertebral disc degeneration, with a range of clinically potentially relevant consequences such as prolapse or herniation,^7^, ^8^, ^9^, ^10^, ^11^, ^12^ disruption of the annulus fibrosus by fibrocartilaginous nucleus pulposus material,^5^ and fibrocartilaginous embolism of the spinal cord with associated ischemic myelopathy.^13^, ^14^, ^15^

Magnetic resonance imaging of the spine has become the method of choice in humans and small companion animals to evaluate intervertebral disc degeneration by applying a 1‐5 MRI degeneration grading scale on T2‐weighted images.^16^, ^17^ Also, sequences have been introduced to evaluate the biochemical composition of the nucleus pulposus.^18^ Accompanying signal changes of the adjacent vertebral endplates and bodies have been evaluated with MRI and described as Modic changes type I (bone edema and inflammation), type II (conversion of the red hematopoietic bone marrow to yellow fat due to bone marrow ischemia), and type III (subchondral bone sclerosis).^19^ These changes have been shown to be related to low back pain in humans.^20^ However, a recent systematic review has stated that associations between Modic changes and low back pain are inconsistent and studies may be biased in a positive associative direction.^21^

Patient size in relation to gantry diameter has so far limited the use of MRI for examination of the lower equine cervical spine and published anatomical and pathological studies are mostly cadaver studies.^22^, ^23^, ^24^, ^25^ Given the fast development of technology and considering the fact that CT of the cervical spine is currently feasible,^26^ MRI of the cervical spine in live horses might not be too far off. Based on our review of the literature, neither magnetic resonance sequences that show equine intervertebral disc degeneration, nor MRI features of the nondegenerated and degenerated equine intervertebral disc have been reported in literature neither proof of concept. Furthermore, no comparison of these features to gross pathology findings has been reported thus far.

The aims of the present study were: (a) to describe the MRI features of nondegenerated and degenerated intervertebral discs in the horse that were macroscopically detectable, (b) to test associations between spinal location and macroscopic degeneration or MRI‐detected annular protrusion and between MRI‐detected annular protrusion and macroscopic degeneration, and (c) to define MRI sequences on which equine cervical intervertebral disc degeneration can be identified. It was hypothesized that MRI‐detected annular protrusion would have a positive association with macroscopic intervertebral disc degeneration.

## 2 MATERIALS AND METHODS

### 2.1 Horses

Horses included in this exploratory methods comparison study were those that had shown clinical signs related to the neck region (such as spinal ataxia, neck pain). All horses were originally presented to the Utrecht University Equine Clinic and had been evaluated by a European College of Veterinary Surgeons‐certified surgeon (W.B.) and/or European College of Equine Internal Medicine‐certified internal medicine specialist (I.D.W.). Thereafter, these horses were included in an ex vivo study on the pathologic aspects of intervertebral disc degeneration published elsewhere.^6^ The current study included only those cases in which MRI scanning and dissection within 4 h of death could be realized. Decisions for subject inclusion or exclusion were made by a European College of Veterinary Diagnostic Imaging‐certified veterinary radiologist (S.V.). Clinical records were reviewed for breed, age, sex, and clinical signs possibly related to the neck region. All horses were client‐owned, owner‐signed informed consent was obtained in accordance with Dutch legislation, and anonymity was preserved.

### 2.2 Data acquisition and interpretation

#### 2.2.1 MRI

Postmortem MRI scans (1.5 T MRI system; Philips Ingenia, Eindhoven, the Netherlands) of the cervical vertebral column in right lateral recumbency were performed after separating the neck from the body between the second and third thoracic vertebrae within 1‐3 h after euthanasia. Magnetic resonance imaging of the complete (n = 9) or partial (n = 2; occiput to C5 and C4 to T2) cervical spines was performed within 4 h following euthanasia. Magnetic resonance imaging scanning times were limited to 2 h as gross, histopathologic, and biochemical examination and sampling was performed afterward. Images were acquired (large anterior coil left lateral aspect, table posterior coil at the right lateral dependent side) mostly in the sagittal plane in accordance with midsagittal dissection afterward. Sequences included sagittal two‐dimensional T1‐weighted turbo SE, two‐dimensional T2‐weighted turbo SE (with and without fat saturation), two‐dimensional proton density turbo SE (with and without fat saturation), and transverse three‐dimensional T1‐weighted turbo field echo. The cartilage sensitive sagittal three‐dimensional water selective cartilage (fast field echo, principle of selected excitation technique, three‐dimensional T1 weighted) and fluid sensitive three‐dimensional water selective fluid (fast field echo, principle of selected excitation technique) sequences were introduced in anticipation of the different intervertebral disc composition in the equine species (Table 1).

**Table 1:** Technical parameters used for MRI studies

**Manufacturer Name of pulse sequence**	**Generic name of pulse sequence**	**TR (ms)**	**TE (ms)**	**Flip angle (degrees)**	**Slice thickness (mm)**	**Interslice (mm)**	**FOV (mm)**	**Matrix**	**Fat sat**	**NSA**
T1W TSE	2D T1W TSE	583	8	90	2.5	−	420 × 190 × 50	600 × 215 (recm960)	−	3
T2W TSE (SPAIR)	2D T2W TSE	3048	124	90	2.5	−	420 × 190 × 50	600 × 208 (672)	− (ProSet)	2
PDW aTSE (SPIR)	2D PDW TSE	4500	30	90	2.27	−	420 × 190 × 50	1008 × 306 (1024)	− (Proset)	2
T1 TFE 3D	3D T1 TFE	8.1	4.6	25	1.2	−	240 × 202 × 155	200 × 166 (432)	TFE 256	4
3D WATSc CLEAR	3D T1 FFE fat sat	20	7.6	25	2.5	−	420 × 190 × 50	620 × 238 (1024)	Proset Fat supp 1331	2
WATSf	3D FFE fat sat	20	8	50	3	−	420 × 187 × 75	852 × 380 (1024)	Proset Fat supp 1331	1

Abbreviations: TR, time of repetition; TE, time of echo; FOV, field of view; Fat Sat, fat saturation; NSA, number of signal averages; T1W, T1‐weighted; TSE, turbo spin echo; SPAIR, spectral attenuated inversion recovery; SPIR, spectral presaturation with inversion recovery; FFE, fast field echo; TFE, turbo field echo; WATSc, water selective cartilage; CLEAR, constant level appearance; WATSf, water selective fluid; Fat supp, fat suppression; Proset, principle of selected excitation technique.

**Figure 1:**
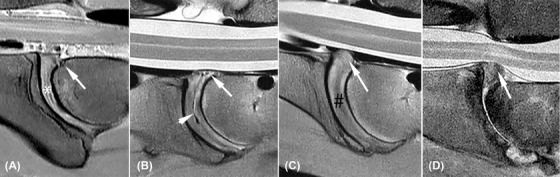
Annular protrusion grading (white arrows) and visibility scoring of the nucleus pulposus; A, Annulus protrusion grade 1, equal to dorsal aspect of vertebral body and visible nucleus pulposus (white asterisk); B, annulus protrusion grade 2, minimal dorsal bulging with respect to vertebral body and gas in the central part of the discus (white arrowhead); C, annulus protrusion grade 3, with moderate dorsal bulging without reaching the spinal cord, (black #); D, severe dorsal bulging of disc material with contact and deformation of the spinal cord

A European College of Veterinary Diagnostic Imaging‐certified veterinary radiologist (S.V.) evaluated the MRI of the cervical intervertebral discs using the available picture archiving and communication system (Impax, AGFA Healthcare, Belgium) during a first evaluation session. During this session the MRI findings in the nondegenerated and degenerated intervertebral discs, as defined by macroscopic assessment, were defined. Sequences per horse were evaluated altogether, with the radiologist not blinded to macroscopic assessment results (nondegenerated or degenerated). Magnetic resonance imaging signal intensity compared to the adjacent muscle signal intensity, signal intensity distribution (dorsal, mid, ventral, rostral, caudal) in the different sequences, and possible visibility of the nucleus pulposus (0, visible; 1, vaguely defined; 2, not visible), intervertebral disc endplate cartilage, vertebral endplate, epiphysis, physis, and spinal cord were descriptively evaluated for midsagittal images (Figure 1). Annular protrusion at C2 to T1 was graded as grade 1 = equal to dorsal aspect of vertebral body, grade 2 = minimal dorsal bulging with respect to vertebral body, grade 3 = moderate dorsal bulging without reaching the spinal cord, and grade 4 = severe dorsal bulging/extrusion of disc material with contact or deformation of the spinal cord (Figure 1). Image quality and detection of anatomic details or changes were evaluated to define MRI sequences that were able to depict equine intervertebral disc degeneration.

A second evaluation session of the MRI images was performed 1 year after the first evaluation. Degeneration status on MRI (non‐degenerated = ND, degeneration = D) was scored by the same radiologist (S.V.), blinded for macroscopic assessment results and horse number. For this, all intervertebral discs per horse were evaluated at once using all available sequences, as would be the same for clinical situations. Criteria for decision‐making were extrapolated from the first session and included (a) the presence of a hypointense signal area in the intervertebral disc in proton density turbo SE sequence, (b) vaguely defined or absent visibility of the nucleus pulposus, and/or (c) presence of a signal void in the intervertebral disc on gradient echo sequences.

Additional findings that were visible on the available MRI and that were potentially attributable to intervertebral disc degeneration were recorded including facet joint pathology (fragmentation or severe enlargement of articular facet), vertebral morphologic variations, and spinal cord signal changes on MRI. Reviewing of available premortem radiographic examinations for pathology including facet joint pathology and vertebral morphologic variations was performed.

#### 2.2.2 Macroscopic assessment

Following MRI scanning the cadaver necks were dissected further and cut along the sagittal midline for macroscopic assessment of the intervertebral discs by a European College of Veterinary Pathologists‐certified pathologist (W.B.), blinded to the MRI findings. The gross examination assessed presence or absence of fibrillation, cleft formation (with gas or associated hemorrhage) and discoloration of the annulus fibrosus and/or nucleus pulposus.^6^ For the purpose of the current study, the intervertebral discs were scored as nondegenerated versus degenerated.

### 2.3 Statistical analysis

Descriptive statistics and statistical analysis were performed by a statistician (H.V.) using statistical analysis freeware (R version 3.4.4, Foundation for Statistical Computing, Vienna, Austria).^27^

Associations between factors spinal location (from C2‐C3 to C7‐Th1) and MRI‐detected annulus protrusion (defined as grades 1 to 4), and outcome macroscopic assessment (defined as non‐degenerated versus degenerated) were assessed with mixed‐effect logistic regression model.^28^ “Horse” (numbers 1‐11) was considered a random effect. The number of observations was small and not all horses had complete data. Spinal location was combined for C2 to C5 versus C5 to T1 and annular protrusion grade 3 and 4 were grouped (grade 3+4) for analysis. Furthermore, annular protrusion grade 2 was also grouped with grade 3+4 (grade 2+3+4) for evaluation. Thereafter, odds ratio and 95% confidence interval were calculated.

## 3 RESULTS

Eleven horses were included (10 Dutch Warmbloods and one Appaloosa; median age: 9 years, range 9 months to 16 years, four mares, one stallion, and six geldings). Clinical recorded signs were spinal ataxia, (severe) neck pain, reduced range of neck motion, and lameness. Horse 10 was presented with an acute onset of severe spinal ataxia following a fall 1 week prior to presentation, but had a history of a minor degree of ataxia for many years (Supporting Information 1).

There were 65 intervertebral discs available for macroscopic evaluation (45 nondegenerated, 20 degenerated; see Table 3) and 60 intervertebral discs for MRI evaluation (42 nondegenerated, 18 degenerated). See Supporting Information 1‐3 for all results per horse, intervertebral disc and for MRI‐degeneration score, and nucleus pulposus visibility.

### 3.1 MRI sequences

The application of mainly sagittal sequences (comparable to midsagittal dissection and enclosing two to three intervertebral discs in one image) with the largest possible field‐of‐view necessitated three times (time consuming) repositioning of the coil and cadaver neck. This resulted in limitation of the imaging protocol to these sequences during this study.

All applied MRI sequences evaluated provided anatomic detail of the intervertebral disc and surrounding structures and all enabled grading of annular protrusion during the first evaluation session. Proton density sequence was essential for intervertebral disc degeneration detection, while T2‐weighted‐turbo SE was least informative due to low contrast resolution of the intervertebral disc structures and surrounding anatomy. Fat saturation proton density and T2‐weighted sequences had both more artifacts and reduced detail. The gradient‐recalled echo sequences (three‐dimensional T1‐turbo field echo, the three‐dimensional water selective cartilage, and three‐dimensional water selective fluid) did show small signal voids due to the presence of gas in clefts or hemorrhage in some intervertebral discs. The three‐dimensional water selective cartilage provided high contrast images of the intervertebral disc and, more important, of the intervertebral disc cartilage endplate. Three‐dimensional water selective fluid was not of additional value.

### 3.2 MRI assessment

#### 3.2.1 MRI descriptive characteristics of macroscopically nondegenerated intervertebral discs (n = 42)

**Figure 2:**
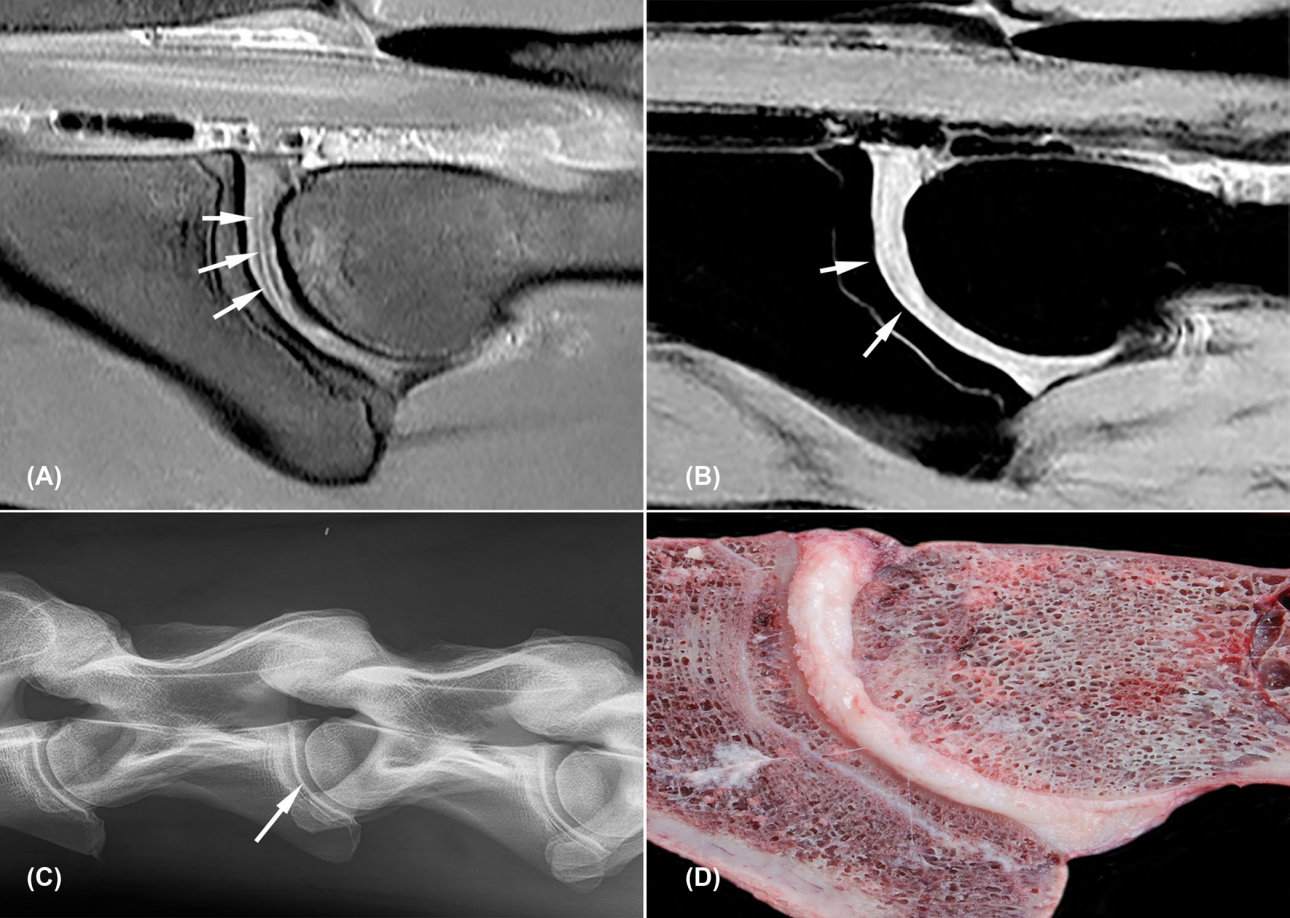
Nondegenerated C3‐C4 intervertebral disc (horse 2). All images are oriented with cranial to the left side of the image and dorsal to the topside of the image. A, Sagittal proton density weighted image; the nucleus pulposus is visible as a very slim hyperintense core surrounded by a thin hypointense rim (white arrows); B, sagittal water selective cartilage image, note the cartilaginous endplate visible as a thin hyperintense line parallel to the vertebral bone surface (white arrows); C, radiograph of C2 to C5 with centrally located intervertebral disc space C3‐C4 (white arrow); D, macroscopic image

The annulus fibrosus was moderately homogeneous isointense compared to surrounding musculature in T1W images with small and vaguely defined hypointense areas in the dorsal and mid‐caudal aspect (Figure 2). The annulus was irregularly and moderately hyperintense compared to musculature on T2‐weighted and proton density (Figure 2A) images with minimally hypointense areas at the dorsal, mid‐caudal, and ventral aspect, and slightly irregular hyperintense on water selective cartilage (Figure 2B). The nucleus pulposus presented as a very slim hyperintense core surrounded by a thin hypointense rim in the cranial mid‐area of the intervertebral disc space in T2‐weighted and proton density images; this was slightly less distinct on water selective cartilage images. On T1‐weighted images only a small hypointense line was visible at the caudal aspect of the nucleus pulposus. The cartilaginous endplates of the intervertebral disc were visible between the annulus fibrosus and vertebral endplates as a separate less than 1 mm thick hyperintense line compared to surrounding structures in water selective cartilage and T1‐weighted images (Figure 2B). The vertebral endplates presented as well‐defined hypointense lines of approximately 1.5‐2 mm thickness in all sequences. They were slightly thinner in the ventral part of the cranial endplate. The epiphyseal areas showed varying degrees of hyperintensity on T1‐weighted, T2‐weighted, and proton density images and were hypointense on fat saturated images, which is consistent with the presence of fatty tissues.

#### 3.2.2 MRI descriptive characteristics of macroscopically degenerated intervertebral discs (n = 18)

**Figure 3:**
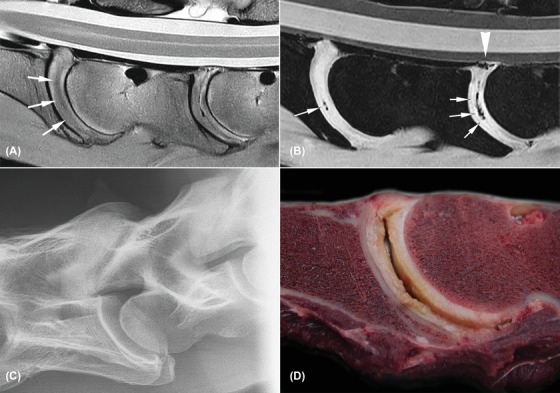
Severely degenerated C5‐C6 and C6‐C7 intervertebral discs with cleft formation (horse 7). All images are oriented with cranial to the left side of the image and dorsal to the topside of the image. A, Sagittal proton density weighted image; diffuse hypointense areas are noted throughout the intervertebral discs with loss of definition of the nucleus (white arrows); B, sagittal water selective cartilage image; signal voids are noted in the clefts (white arrows) and dorsal aspect at the dorsal longitudinal ligament of C6‐C7 (white arrow head); C, radiograph of C6‐C7 depicts moderate degenerative joint disease but no changes at the intervertebral disc space; D, macroscopic image of C6‐7 with a large cleft centrally, fibrillation, and yellow discoloration. The dorsal longitudinal ligament tear is not visible in this image

**Figure 4:**
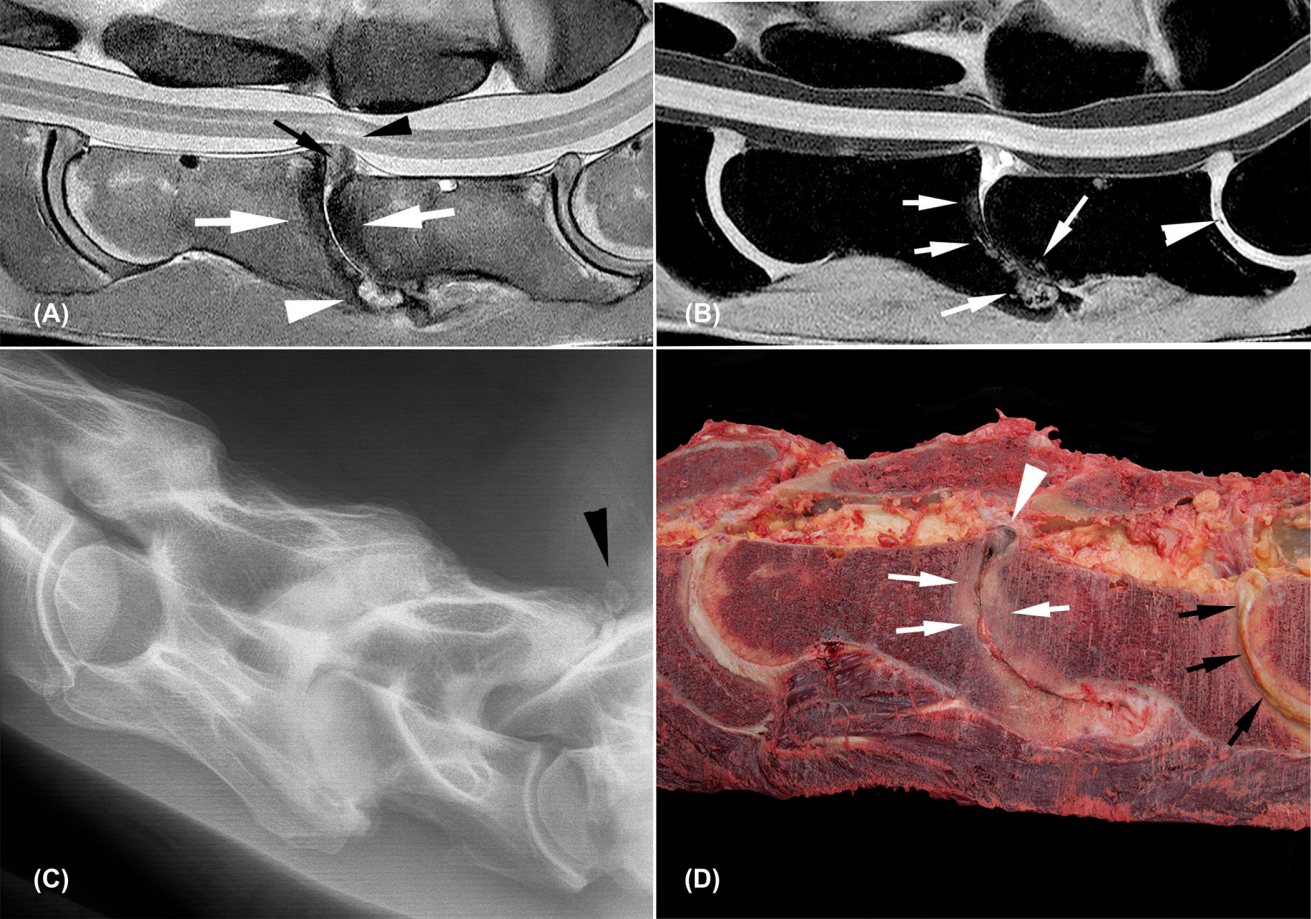
Severely degenerated C6‐C7 and C7‐T1 intervertebral discs with complete collapse of C6‐C7 (horse 10). All images are oriented with cranial to the left side of the image and dorsal to the topside of the image. A, Sagittal proton density weighted image; vertebral endplates of C6‐C7 are diffusely hypointense (white arrows) with a hyperintense area ventrally (arrowhead) and grade 4 annulus protrusion of disc material (black arrow) with adjacent intra‐medullary hyperintense signal (black arrowhead) and dorsal displacement of the spinal cord. C5‐C6 and C7‐T1 intervertebral discs show diffuse hypointense areas with grade 1 and grade 3 annulus protrusion. B, Sagittal water selective cartilage image; C6‐C7 irregular hyperintense areas are seen in the vertebral endplates (white arrows) and hyperintense signal at the remaining disc material. Small signal voids are noted in the intervertebral disc of C7‐T1 (white arrowhead); C, radiograph shows complete collapse with severe remodeling of the vertebral endplates at C6‐C7 together with severe degenerative joint disease and mild ventral subluxation of C7. Separate mineralizations are noted dorsal to the facet joints of C7‐T1 (black arrowhead) and the intervertebral disc space is slightly narrower compared to C5‐C6 intervertebral disc space; D, macroscopic image; severe remodeling of the vertebral endplates (white arrows), dark discoloration of the dorsal protruding disc material at C6‐C7 (white arrowhead). Severe cleft formation, fibrillation, and yellow discoloration of the C7‐T1 intervertebral disc (black arrows)

The annulus fibrosus contained moderately patchy to diffuse hypointense areas, which were subjectively most clearly defined in proton density images (Figures 3 and 4). These areas were more distinct and expanded further in dorsal and ventral directions compared to the above‐described small and vaguely defined hypointense areas in the dorsal and mid‐caudal aspect of the normal intervertebral discs.

The delineation of the nucleus pulposus was irregular vaguely defined or completely absent (Supporting Information 2 and 3). Gradient‐recalled echo sequence signal voids and/or susceptibility artifacts were seen mostly in the center of the intervertebral disc (at the level of the nucleus pulposus), consistent with bleeding and/or gas in a macroscopically ruptured intervertebral disc. Signal voids were seen in the dorsal aspect of the dorsal annulus toward the vertebral canal in one C6‐C7 intervertebral disc (Figure 3b). The vertebral structures, intervertebral disc endplate cartilage, and spinal cord were comparable to the previously described nondegenerated intervertebral discs in all cases except one horse (horse 10; Figure 4). In this specific case, a complete collapse with associated dorsal extrusion and protrusion of disc material (annulus protrusion grade 4) and minimal vertebral subluxation due to tilting of the cranial aspect of C7 was noted. Severe shape and signal changes of the vertebral endplates and bodies were present showing as patchy hypointense signal on all sequences and varying from vaguely defined hyperintense areas in fat saturated proton density to more well‐defined areas on Water selective cartilage images. The spinal cord dorsal to this intervertebral disc had an ill‐defined hyperintense signal on T2‐weighted and proton density images (Figures 4a and 4b). Gross pathological examination showed a complete collapse of the intervertebral disc space with severe remodeling of the vertebral endplates and bodies and greenish extruded disc material associated with hemorrhage in the vertebral canal (Figure 4d).

#### 3.2.3 MRI annular protrusion grading

Annular protrusion (grade 2 to 4) was present in 26 of 30 caudal (C5 to T1) versus 13 of 31 cranial (C2 to C5) intervertebral discs. No (grade 1), minimal (grade 2), moderate (grade 3), and severe (grade 4) annulus protrusion was seen in 22, 29, nine, and one intervertebral discs, respectively (Table 2).

**Table 2:** Frequency distribution of MRI‐detected annulus protrusion grade 1‐4 per spinal location (C2‐C3 to C7‐T1)

	MRI‐annulus protrusion grade			
	1	2	3	4
All discs	21	29	9	1
C2‐C3	7	3	0	0
C3‐C4	6	2	2	0
C4‐C5	4	5	1	0
C5‐C6	1	8	1	0
C6‐C7	0	6	3	1
C7‐T1	3	5	2	0

#### 3.2.4 MRI degeneration status grading

Degeneration grading performed during the second evaluation session resulted in 37 intervertebral discs being scored as nondegenerated and 24 intervertebral discs scored as degenerated (See Supporting Information 2 and 3). Discrepancy of the MRI degeneration grading with macroscopic assessment was noted in 16 (MRI grade 11 degenerated, five nondegenerated) intervertebral discs of 60 evaluated discs.

### 3.3 Macroscopic assessment

Macroscopic assessment was performed in 65 discs (of which 60 intervertebral discs were also evaluated with MRI) and not possible in one C4‐C5 intervertebral disc (Supporting Information 1), which was excluded from further statistical analysis. Of the discs evaluated with MRI, 42 intervertebral discs were judged as nondegenerated and 18 as degenerated, with most of the degenerated discs located at C6‐C7 (5/18) and C7‐T1 (5/18). It was remarkable that gross degeneration of all intervertebral discs was observed in one horse (horse 6), while in two other horses (horses 1 and 4) none of the intervertebral discs showed signs of gross degeneration. Intervertebral disc degeneration was detected in 13 of 30 caudal (C5 to T1) versus five of 30 cranial (C2 to C5) intervertebral discs. On gross pathological examination, hemorrhage and tearing of the dorsal longitudinal ligament was noted at a degenerated and torn intervertebral disc at C6‐C7 in one Dutch Warmblood (horse 7) with a grade 3 annular protrusion without changes in shape or signal of the spinal cord, vertebral endplates, or intervertebral disc space width on MRI.

#### 3.3.1 Additional MRI and radiographic findings

Radiography premortem was available in nine horses, with the following additional findings. Severe deformation of a right‐sided C5‐C6 facet joint due to osteochondrosis, bilaterally severely enlarged facet joints at C6‐C7, and axially positioned rounded, focal, mineralized areas with enlargement of the left‐sided facet joint of C7‐T1 were found in one horse (horse 5). Intervertebral discs of C5‐C6 to C7‐T1 showed macroscopic signs of degeneration in this horse. A rounded, solitary area of dystrophic mineralization was present in the soft tissue axial to the first left rib head with severe intervertebral disc degeneration of C7‐T1 in one horse (horse 7). Horse 10 that showed complete collapse of the intervertebral disc space had severe facet joint arthrosis at C6‐7 and bilateral mineralisation in the soft tissue dorsal to C7‐T1. Morphologic variations, more specifically a shift of the transverse process ventral lamina of C6 to C7, were seen on the sagittal MRI images in four Dutch Warmblood horses (36%); bilateral in two (horses 2 and 11) and unilateral in the other two (horses 4 and 6). Macroscopic intervertebral disc degeneration was found in these four horses and involved the intervertebral discs at C6‐7 and C7‐T1 in two and three horses, respectively.

### 3.4 Statistical analysis

Mixed‐effect logistic regression modeling with “horse” as random effect was performed (Table 3).

**Table 3:** Odds ratio (95% confidence interval) of the univariable mixed‐effect logistic regression model for macroscopic degeneration status with MRI‐detected annulus protrusion and spinal location in 11 horses

	Macroscopy Normal n (%)	Macroscopy Degenerated n (%)	OR (95% CI)
Annulus (n = 60)	42 (70.0)	18 (30.0)	
Grade 1 (Ref.)	19 (45.2)	2 (11.1)	1.0
Grade 2	19 (45.2)	10 (55.6)	6.5 (0.91‐45.9)
Grade 3–4	4 (9.5)	6 (33.3)	40.6 (2.1‐776.6)
Location (n = 65)	45 (69.2)	20 (30.8)	
C2‐C3 (Ref.)	10 (22.2)	1 (5.0)	1.0
C3‐C4	10 (22.2)	1 (5.0)	1.0 (0.02‐63.1)
C4‐C5	7 (15.6)	3 (15.0)	10.6 (0.3‐396.8)
C5‐C6	10 (22.2)	1 (5.0)	1.0 (0.02‐63.1)
C6‐C7	4 (8.9)	7 (35.0)	143.8 (2.6‐7869.1)
C7‐T1	4 (8.9)	7 (35.0)	143.8 (2.6‐7869.1)
Location (n = 65)	45 (69.2)	20 (30.8)	
C2‐C5 (Ref.)	27 (60.0)	5 (25.0)	1.0
C5‐T1	18 (40.0)	15 (75.0)	7.1 (1.6‐30.8)

Abbreviations: OR, odds ratio; CI, confidence interval; Ref., reference.

The odds to have macroscopic degeneration with annular protrusion grade 2 was higher than the odds to have annular protrusion grade 1 and for grade 3+4 even higher with a very wide confidence interval indicative for the small data set (Table 3). A statistically significant association between annular protrusion and macroscopic intervertebral disc degeneration was found by applying the mixed‐effect model for annular protrusion grade 1 versus grade 2+3+4 (OR 8.1, 95% confidence interval, 1.3‐50.5).

Macroscopic degeneration was found to be statistically significantly associated with spinal location (Table 3). Spinal location C4‐C5, C6‐C7, and C7‐T1 showed an increased risk for macroscopic degeneration compared to C2‐C3, although the confidence intervals are very wide. Location was thereafter recoded on cranial (C2 to C5) versus caudal (C5 to T1), resulting in higher odds to find macroscopic degeneration in the caudal than cranial cervical vertebral column (odds ratio 7.1, 95% confidence interval, 1.6‐30.8). The model including both spinal location (C5‐T1 vs C2‐C5) and annular protrusion (3+4 vs 1 and 2) did not result in statistically significant associations with macroscopic degeneration (odds ratio = 3.1, 95% confidenc interval 0.6‐16.1 and odds ration = 4.3, 95% confidence interval, 0.6‐32.7, respectively).

## 4 DISCUSSION

Based on our review of the literature, this is the first published study to describe ex vivo MRI findings in nondegenerated and degenerated equine intervertebral discs. The study provides proof‐of‐concept of the potential for using MRI to depict macroscopically detectable equine intervertebral disc degeneration. Nucleus and annulus degeneration can be detected in proton density images; annulus protrusion and/or extrusion can be detected in all images. Cleft formation in the disc, within some instances the associated hemorrhage and/or gas collection, can be detected in gradient‐recalled echo sequences such as water selective cartilage. Results from the second evaluation session demonstrate, however, some discrepancy in degeneration grading (nondegenerated vs degenerated) between MRI and macroscopic assessment in 16 of 60 intervertebral discs. Discrepancy was greatest for discs assessed as nondegenerated by macroscopy but degenerated with MRI, which can indicate a need to further specify MRI features of disc degeneration. From this study, it appeared that grade 2 and 3‐4 of MRI‐detected annular protrusion did have higher odds than grade 1 to have intervertebral disc degeneration on gross pathology. Macroscopic degeneration (n = 18) was statistically significantly associated with the spinal location with a wide confidence interval as described before.^6^ It should be realized that macroscopic degeneration was seen in 13 of 30 caudal (C5 to T1) versus five of 30 cranial (C2 to C5) intervertebral discs that were also evaluated by MRI.

The results of our study show that the nucleus pulposus dehydration‐based T2 sequences as applied in humans and dogs^16^, ^17^ are most likely less suited for MRI evaluation of the equine intervertebral disc. This is probably due to the fact that the annular and nucleus part in this species consist of fibrous and cartilaginous tissues and were irregularly hypointense on T2 sequence while being more consistent in signal intensity in proton density images.^6^ Visibility of the nucleus pulposus in proton density and T2 sequences was reduced in macroscopically degenerated intervertebral discs (Supporting Information 2 and 3). This might be caused by diffuse fibrillation and therefore structural changes of both fibrous as well as cartilaginous tissues as scored with macroscopy.

The current study used mainly routine (proton density, T2‐weighted, and T1‐weighted) MRI sequences in the sagittal plane. For further evaluation of adjacent anatomic structures such as exiting nerves, transverse or dorsal images should be included. However, these were considered outside the scope of this study and were not performed due to limited scanning time. It should however be considered that other sequences in different planes perform better in detection of different aspects of equine disc degeneration, such as lateralized annular protrusion or early stage of degeneration. An in‐depth MRI study of histopathological confirmed degenerated discs, using different sequences and imaging planes is needed to gain further insight. This being said, histopathology results of intervertebral disc degeneration have not been conclusive yet and the macroscopic appearance has been more discriminative so far.^6^ Furthermore, both in dogs and humans histological scoring of intervertebral disc degeneration is validated by macroscopic grading among others.^29^ This might also explain the discrepancies between the macroscopic and MRI assessments of intervertebral disc degeneration during the second evaluation session and may reflect the fact that MRI makes use of the biochemical composition of tissues for image formation.

The susceptibility of the gradient‐recalled echo sequence as can be seen with cleft formation or tearing of the intervertebral disc proved to add useful information. The presence of gas due to vacuum phenomenon is well known in disc degeneration and can be helpful in identifying degenerated intervertebral discs.^30^ Water selective cartilage sequence (brand‐specific) is a less common MRI sequence that was previously reported to be excellent for cartilage evaluation.^31^ Equivalent sequences exist in other high‐field MRI systems and are known as fat saturated three‐dimensional T1‐weighted fast field echo, or fast‐low angle shot. Water selective cartilage provided good means to evaluate the intervertebral disc cartilaginous endplate, a structure thought to play a crucial role in nutrient transport to the intervertebral disc.^3^

Equine vertebral morphologic variations, vertebral pathology, and their clinical manifestations have previously been described.^32^, ^33^ The small group of horses in this study presented varying clinical signs related to the cervical area. The severe spinal ataxia of the horse with the collapsed intervertebral disc (horse 10) and associated changes to the spinal cord were considered a direct consequence of the macroscopically visible intervertebral disc extrusion. The tear in the dorsal longitudinal ligament and hemorrhage in concurrence with a degenerated disc in the horse with low neck pain and ataxia (horse 7) were also considered causative. A low volume‐high velocity disc extrusion with fibrocartilaginous embolism might have caused spinal cord damage in this horse.^14^ Other relationships of the recorded MRI and macroscopic degenerative findings to the clinical signs are possible, but need further in‐depth clinical studies. The clinical manifestation of low back pain in humans with lumbar intervertebral disc degeneration varies greatly between individuals.^20^ In addition to intervertebral disc degeneration, pain also appears to correlate inconsistently with vertebral Modic changes grade 1 (bone edema and inflammation) and discal endplate lesions.^19^, ^20^, ^21^ In the current study, Modic changes or vertebral endplate lesions were not observed except in the horse with the completely collapsed intervertebral disc space at C6‐C7 (horse 10).

Early intervertebral disc degeneration in humans and small companion animals is mostly characterized by loss of proteoglycans and therefore loss of osmotic pressure and hydration in both nucleus pulposus and annulus fibrosis.^34^ In a later stage, replacement of type II‐collagen fibers with type I‐collagen fibers occurs.^34^ Both events provoke changes in mechanical and tensile properties, which can cause morphological changes such as decreased intervertebral disc width and protrusion and/or extrusion due to tearing of the annulus.^34^

Annular protrusion detected with MRI shows an association with macroscopic intervertebral disc degeneration, even in this small group of horses. This is potentially of great clinical relevance, as focal disruption of the annulus fibers with fibro‐cartilage has been described in horses as a possible cause of protrusion, fibrocartilaginous embolism, or intervertebral disc collapse with varying clinical complications.^5^, ^14^ Annulus protrusion and spinal cord diameter can be evaluated by the currently available in vivo imaging methods such as myelography or vertebral canal endoscopy.^35^, ^36^ However, these techniques are more invasive and will not provide additional information of changes in the intervertebral disc, spinal cord, nerve roots, and vertebrae. This study shows as proof‐of‐concept that MRI can offer an important additional benefit and that further application for the diagnosis of intervertebral disc degeneration in horses needs to be further investigated.

To date, published reports on MRI of the equine cervical spine in live horses are very scarce due to the technical limitations, but it can be expected that these will be overcome in the future.

It is concluded that macroscopically confirmed degeneration in the equine cervical intervertebral disc can be detected with MRI and the best sequence to do so is the proton density turbo SE in combination with a gradient‐recalled echo sequence. Furthermore, intervertebral disc degeneration was noted to be associated to caudal spinal location; annular protrusion on MRI had higher odds to have macroscopic degeneration of the equine cervical intervertebral disc. Magnetic resonance imaging of the equine cervical spine is a still scarcely available but promising technique. Further research is needed to improve practical feasibility and determine its full clinical potential in unraveling the cervical pathology in horses and hence the benefits of its clinical application.

## LIST OF AUTHOR CONTRIBUTIONS

### Category 1

(a) Conception and Design: Veraa, Bergmann, Wijnberg, Back, van den Belt
(b) Acquisition of Data: Veraa, Bergmann, Wijnberg, Back.
(c) Analysis and Interpretation of Data: Veraa, Bergmann, Vernooij, Nielen

### Category 2

(a) Drafting the Article: Veraa
(b) Revising Article for Intellectual Content: Veraa, Bergmann, Wijnberg, Back, Vernooij, Nielen, van den Belt

### Category 3

(a) Final Approval of the Completed Article: Veraa, Bergmann, Wijnberg, Back, Vernooij, Nielen, van den Belt

## CONFLICT OF INTEREST

The authors declare no conflict of interest.

## Supporting information

Supplement 1: Tabular overview of the macroscopic evaluation and MRI‐detected annulus protrusion grades (1‐4) available per intervertebral disc, for all horses included in the study (n = 11).
Supplement 2: Tabular overview of MRI nucleus pulposus visibility (n = 0‐2) and MRI degeneration scoring available per intervertebral disc, for all horses included in the study (n = 11).
Supplement 3: Tabular overview of macroscopic degeneration scoring (0‐5) (Bergmann et. al, 2018) versus MRI degeneration scoring (ND‐D) and visibility of the nucleus pulposus scoring (0,1‐2).
