# Supplementary material for: Equine cervical intervertebral disc degeneration is associated with location and MRI features
Source: Vet Radiol Ultrasound. 2019 Jul 28;60(6):696–706. doi: 10.1111/vru.12794 (PMC6899552; doi:10.1111/vru.12794)
Supplement: Supplementary file 1 — Supplement 1: Tabular overview of the macroscopic evaluation and MRI‐detected annulus protrusion grades (1‐4) available per intervertebral disc, for all horses included in the study (n = 11). Supplement 2: Tabular overview of MRI nucleus pulposus visibility (n = 0‐2) and MRI degeneration scoring available per intervertebral disc, for all horses included in the study (n = 11). Supplement 3: Tabular overview of macroscopic degeneration scoring (0‐5) (Bergmann et. al, 2018) versus MRI degeneration scoring (ND‐D) and visibility of the nucleus pulposus scoring (0,1‐2). [file VRU-60-696-s001.docx]

Supplement 1: Tabular overview of the macroscopic evaluation and MRI-detected annulus protrusion grades (1-4) available per intervertebral disc, for all horses included in the study (n=11).

| **Horse** | **Age (yr)** | **Gender** | **Breed** | **Clinical signs** | **C2-C3** | | **C3-C4** | | **C4-C5** | | **C5-C6** | | **C6-7** | | **C7-T1** | |
| --- | --- | --- | --- | --- | --- | --- | --- | --- | --- | --- | --- | --- | --- | --- | --- | --- |
|  |  |  |  |  | m | a | m | a | m | a | m | a | m | a | m | a |
| 1 | 0.8 | Stallion | RDSH | Severe ataxia | ND | 1 | ND | 1 | N/A | 1 | ND | 1 | ND | 2 | ND | 1 |
| 2 | 5 | Mare | RDSH | Lameness, neck pain | ND | 1 | ND | 2 | ND | 2 | ND | 2 | D | 2 | ND | 2 |
| 3 | 6 | Gelding | RDSH | Ataxia | ND | 1 | ND | 1 | D | 2 | ND | 2 | ND | 2 | D | 2 |
| 4 | 7 | Mare | RDSH | Chronic lameness, stiff neck, tripping | ND | 1 | ND | 1 | ND | 1 | ND | 2 | ND | 3 | ND | 2 |
| 5 | 9 | Mare | RDSH | Stiff neck, neck pain, lameness | ND | 1 | ND | 1 | ND | 1 | ND | 2 | D | 2 | D | 1 |
| 6 | 11 | Gelding | RDSH | Ataxia | D | 2 | D | 2 | D | 2 | D | 3 | D | 3 | D | 2 |
| 7 | 16 | Gelding | RDSH | Lame, neck pain, ataxia | ND | 1 | ND | 1 | D | 1 | ND | 2 | D | 2 | ND | 1 |
| 8 | 7 | Gelding | RDSH | Lame, resistance, neck pain | ND | 2 | ND | 3 | ND | 3 | ND | 2 | ND | 2 | D | 3 |
| 9 | 9 | Mare | RDSH | Resistance, neck muscle atrophy, neck pain | ND | 1 | ND | 1 | ND | 1 | ND | 2 | D | 3 | D | 2 |
| 10 | 11 | Gelding | Appaloosa | Ataxia | ND | N/A | ND | N/A | ND | 2 | ND | 2 | D | 4 | D | 3 |
| 11 | 15 | Gelding | RDSH | Ataxia, neck pain | ND | 2 | ND | 3 | ND | 2 | ND | N/A | D | N/A | D | N/A |

Notes: yr, year; RDSH, Royal Dutch Sport Horse; m, macroscopy; a, annulus grade; ND, non-degenerated; D, degenerated; N/A= not available.

Supplement 2: Tabular overview of MRI nucleus pulposus visibility (n=0-2) and MRI degeneration scoring available per intervertebral disc, for all horses included in the study (n=11).

| **Horse** | **C2-C3** | | | **C3-C4** | | | **C4-C5** | | | **C5-C6** | | | **C6-7** | | | **C7-T1** | | |
| --- | --- | --- | --- | --- | --- | --- | --- | --- | --- | --- | --- | --- | --- | --- | --- | --- | --- | --- |
|  | m | n | d | m | n | d | m | n | d | m | n | d | m | n | d | m | n | d |
| 1 | ND | 1 | ND | ND | 0 | ND | N/A | 0 |  | ND | 0 | ND | ND | 0 | ND | ND | 0 | ND |
| 2 | ND | 0 | ND | ND | 0 | ND | ND | 0 | ND | ND | 0 | ND | D | 2 | D | ND | 2 | D |
| 3 | ND | 0 | ND | ND | 0 | ND | D | 0 | ND | ND | 0 | ND | ND | 0 | D | D | 1 | D |
| 4 | ND | 0 | ND | ND | 0 | ND | ND | 0 | ND | ND | 0 | ND | ND | 0 | ND | ND | 0 | ND |
| 5 | ND | 0 | D | ND | 0 | ND | ND | 0 | ND | ND | 0 | ND | D | 1 | D | D | 1 | ND |
| 6 | D | 1 | D | D | 1 | ND | D | 1 | ND | D | 1 | D | D | 1 | D | D | 1 | ND |
| 7 | ND | 0 | D | ND | 0 | D | D | 0 | D | ND | 1 | D | D | 0 | D | ND | 1 | D |
| 8 | ND | 0 | D | ND | 0 | ND | ND | 0 | ND | ND | 0 | ND | ND | 2 | D | D | 1 | D |
| 9 | ND | 0 | ND | ND | 0 | ND | ND | 0 | ND | ND | 0 | ND | D | 1 | D | D | 2 | D |
| 10 | ND |  |  | ND |  |  | ND | 1 | ND | ND | 1 | D | D | 2 | D | D | 1 | D |
| 11 | ND | 0 | ND | ND | 0 | D | ND | 1 | ND | ND |  |  | D |  |  | D |  |  |

Notes: m=macroscopic assessment (ND=non-degenerated, D=degenerated), n= MRI nucleus visibility (0=visible, 1=vaguely defined, 2=not visible) ; d= MRI degeneration scoring (ND= non-degenerated, D= degenerated)

Supplement 3: Tabular overview of macroscopic degeneration scoring (0-5) (Bergmann et. al, 2018) versus MRI degeneration scoring (ND-D) and visibility of the nucleus pulposus scoring (0,1-2).

| **Macroscopic assessment degeneration score** | **MRI degeneration scoring (0-1)** | | **MRI nucleus pulposus visibility scoring (0,1-2)** | | |
| --- | --- | --- | --- | --- | --- |
|  | **ND** | **D** | **0** | **1** | **2** |
| 1 | 13 | 3 | 13 | 2 | 1 |
| 2 | 18 | 8 | 21 | 4 | 1 |
| 3 | 0 | 1 | 0 | 1 | 0 |
| 4 | 5 | 6 | 2 | 8 | 1 |
| 5 | 0 | 6 | 1 | 3 | 2 |

Notes: ND= non-degenerated, D= degenerated
